# A live attenuated vaccine prevents replication and transmission of H7N9 highly pathogenic influenza viruses in mammals

**DOI:** 10.1038/s41426-018-0154-6

**Published:** 2018-09-12

**Authors:** Wenyu Yang, Xin Yin, Lizheng Guan, Mei Li, Shujie Ma, Jianzhong Shi, Guohua Deng, Yasuo Suzuki, Hualan Chen

**Affiliations:** 1 College of Veterinary Medicine, Gansu Agriculture University, 730030 Lanzhou, China; 2 State Key Laboratory of Veterinary Biotechnology, Harbin Veterinary Research Institute, Chinese Academy of Agricultural Sciences, 150001 Harbin, China; 3 College of Life and Health Sciences, Chubu University, Health Science Hills, 1200 Matsumoto-cho Kasugai-Shi, Aichi, 487-8501 Japan

## Abstract

H7N9 influenza viruses emerged in 2013 and have caused severe disease and deaths in humans in China. Some H7N9 viruses circulating in chickens have mutated to highly pathogenic viruses that have caused several disease outbreaks in chickens. Studies have shown that when the H7N9 highly pathogenic viruses replicate in ferrets or humans, they easily acquire certain mammalian-adapting mutations and become highly lethal in mice and highly transmissible in ferrets by respiratory droplet, creating the potential for human-to-human transmission. Therefore, the development of effective control measures is a top priority for H7N9 pandemic preparedness. In this study, we evaluated the protective efficacy of a cold-adapted, live attenuated H7N9 vaccine (H7N9/AAca) against two heterologous H7N9 highly pathogenic viruses in mice and guinea pigs. Our results showed that one dose of the H7N9/AAca vaccine prevented disease and death in mice challenged with two different H7N9 highly pathogenic viruses, but did not prevent replication of the challenge viruses; after two doses of H7N9/AAca, the mice were completely protected from challenge with A/chicken/Hunan/S1220/2017(H7N9) virus, and very low viral titers were detected in mice challenged with H7N9 virus CK/SD008-PB2/627 K. More importantly, we found that one dose of H7N9/AAca could efficiently prevent transmission of CK/SD008-PB2/627 K in guinea pigs. Our study suggests that H7N9/AAca has the potential to be an effective H7N9 vaccine and should be evaluated in humans.

## Introduction

Since the H7N9 influenza viruses emerged in February 2013, they have caused 1567 human infections in China, 615 of which were fatal^1^. The 2013 H7N9 viruses isolated from birds could replicate and transmit efficiently in chickens but were nonpathogenic^2^. Avian and human H7N9 viruses were able to bind both avian-type and human-type receptors^3–6^, and the human isolates were more lethal in mice and transmitted more efficiently in ferrets than the avian isolates^2^, mainly because the viruses acquired mutations in the basic polymerase 2 (PB2) gene (PB2 627 K or PB2 701 N) during their replication in humans^7,8^. In early 2017, H7N9 viruses carrying a four-amino-acid insertionin the cleavage site of the hemagglutinin (HA) gene were detected in samples collected from live poultry markets in Guangdong province, and animal studies indicated that these HA mutants were highly pathogenic for chickens^9,10^. Studies have revealed that the H7N9 HA mutants could acquire additional mutations during their replication in ferrets or humans, and then become highly lethal in mice and ferrets and transmissible in ferrets via respiratory droplet^10,11^. Yang et al. reported that 50% of human cases of infection with the highly pathogenic H7N9 influenza viruses were fatal^12^. These facts demonstrate that the H7N9 highly pathogenic influenza viruses pose an increased threat to humans.

Vaccination is a key strategy for human influenza prevention and control, and different kinds of vaccines against the H7N9 influenza virus have been developed and tested in humans and animals^13–20^. We previously generated a live attenuated H7N9 vaccine seed virus, termed H7N9/AAca that bears the HA and neuraminidase (NA) gene segments from A/Anhui/1/2013 (H7N9) (AH/1)and its remaining six gene segments from the cold-adapted influenza virus A/Ann Arbor/6/60 (H2N2), by using plasmid-based reverse genetics. Animal studies indicated that H7N9/AAca was attenuated in mice and ferrets, and induced robust neutralizing antibody responses in mice, ferrets, and guinea pigs^21^. Mice and ferrets immunized with two doses were completely protected from homologous AH/1 challenge, and guinea pigs vaccinated with only one dose were fully protected from transmission when exposed to or in contact with H7N9 virus-inoculated animals^21^.The AH/1 virus transmits efficiently in ferrets and guinea pigs via respiratory droplet^2,21^, but causes mild symptoms inmammals compared with those caused by the H7N9 highly pathogenic viruses carrying the PB2 627 K or 701 N mutations^10,12,22,23^. It is not known whether this H7N9/AAca vaccine provides protection against the H7N9 highly pathogenic viruses in mammals. Therefore, in this study, we evaluated the protective efficacy of the H7N9/AAca vaccine against two heterologous H7N9 highly pathogenic viruses in mice and guinea pigs.

## Results

### Genetic and antigenic differences between the 2013 AH/1 virus and the 2017 H7N9 highly pathogenic viruses

We used two H7N9 highly pathogenic influenza viruses as challenge strains to evaluate the protective efficacy of the H7N9/AAca vaccine in mice. The CK/SD008-PB2627K virus is a ferret-adapted H7N9 virus that is highly lethal in mice, with a 50% mouse lethal dose (MLD_50_) value of 10^1.8^ 50% egg infectious dose (EID_50_)^10^, and A/chicken/Hunan/S1220/2017(H7N9) (CK/S1220) is a naturally isolated H7N9 virus that is highly lethal in mice (MLD_50_ = 10^3.2^EID_50_) but does not have the PB2 627 K or 701 N mutations^24^.The HA and NA genes of these two lethal viruses share over 97% identity with those of the H7N9/AAca surface gene donor virus AH/1(Table 1). Antisera generated in ferrets against AH/1 virus and H7N9/AAca cross-reacted with the two H7N9 highly pathogenic viruses, but the titers to the CK/SD008-PB2/627 K and CK/S1220 viruses were 2- and 8-fold lower than the homologous titers, respectively (Table 1).

**Table 1:** Genetic and the antigenic relationships between the A/Anhui/1/2013(AH/1) virus and two H7N9 highly pathogenic influenza viruses

Virus	Gene identity (%)				Cross-reactive HI antibody titers of ferret antisera induced by different viruses	
	Hemagglutinin		Neuraminidase			
	Nucleotide	Amino acid	Nucleotide	Amino acid	AH/1^a^	H7N9/AAca^b^
A/Anhui/1/2013 (H7N9)(AH/1)	100	100	100	100	640	160
H7N9/AAca	100	100	100	100	320	160
A/chicken/Hunan/S1220/2017(H7N9)(CK/SD1220)	97.9	97.1	97.9	97.2	80	20
CK/SD008-PB2/627K	98.5	97.9	98.6	98.3	320	80

^a^The sera were collected in the previously study by Zhang et al.^2^. Ferrets were inoculated intranasally with 10^6^EID_50_ of the AH/1 virus and were euthanized for serum collection three weeks post-inoculation

^b^The sera were collected in the previously study by Kong et al.^21^. Ferrets were inoculated intranasally with one dose of 10^6^EID_50_ of the live attenuated vaccine H7N9/AAca, and serum samples were collected from the ferrets three weeks post-inoculation

### The H7N9/AAca vaccine protects mice against challenge with different heterologous H7N9 highly pathogenic viruses

Groups of 10 six-week-old female BALB/c mice were anesthetized with CO_2_ and vaccinated intranasally (i.n.) with one or two doses (three weeks apart) of 10^6^ EID_50_ of H7N9/AAca in 50 µl or with 50 µl of PBS (mock). Sera were collected on day 21 post-vaccination (p.v.) for hemagglutinin inhibition (HI) and neutralization (NT) antibody detection.The mice were then challenged i.n. with 100 MLD_50_ of the CK/S1220 (10^5.2^ EID_50_) or CK/SD008-PB2/627 K (10^3.8^ EID_50_) virus. Nasal turbinates, lungs, spleens, kidneys, and brains were collected from three mice in each group on day 3 post-challenge (p.c.) for virus titration; the remaining seven mice in each group were observed daily for body weight change and death for a total of two weeks.

**Fig. 1:**
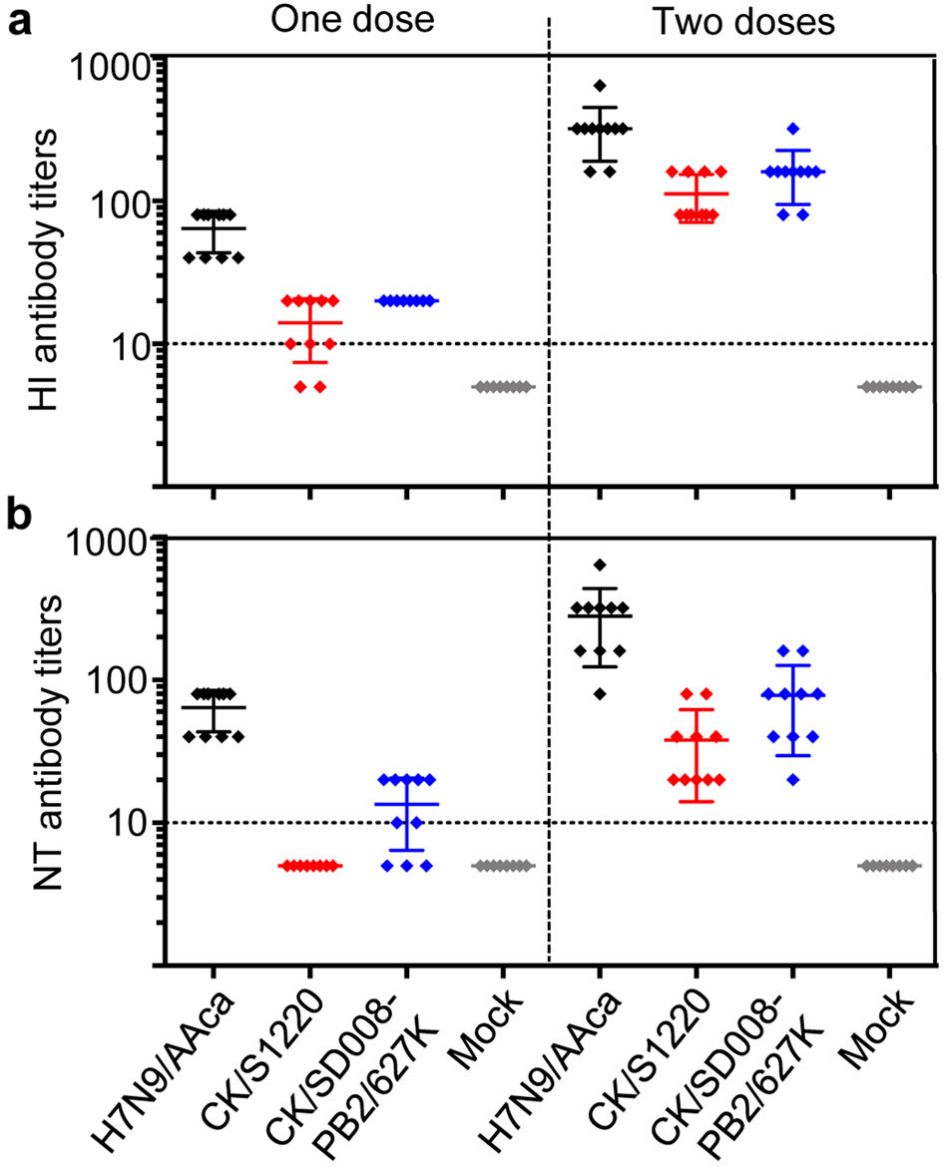
Antibody titers induced in mice by H7N9/AAca against different viruses. Groups of ten mice were inoculated with one or two doses, with a 3-week interval, of 10^6^ EID_50_ of the H7N9/AAca virus vaccine. Three weeks after the last dose, sera were collected to determine HI (**a**) and NT (**b**) antibody titers by using the homologous H7N9/AAca virus and the heterologous CK/S1220 and CK/SD008-PB2/627 K viruses, respectively. The dotted lines indicate the lower limit of detection

The mean HI antibody titers against H7N9/AAca, CK/S1220, and CK/SD008-PB2/627 K were 65, 13, and 20, respectively, in the single dose vaccinated groups, and were 320, 110, and 160, respectively, in the groups that received two doses of vaccine (Fig. 1a). The mean NT antibody titers against the H7N9/AAca, CK/S1220, and CK/SD008-PB2/627 K viruses were 56, undetectable, and 12, respectively, in the single dose vaccinated groups, and 280, 40, and 80, respectively, in the groups that received two doses of vaccine (Fig. 1b). These results indicate that there are slight antigenic difference between the vaccine strain and the highly pathogenic H7N9 viruses.

**Fig. 2:**
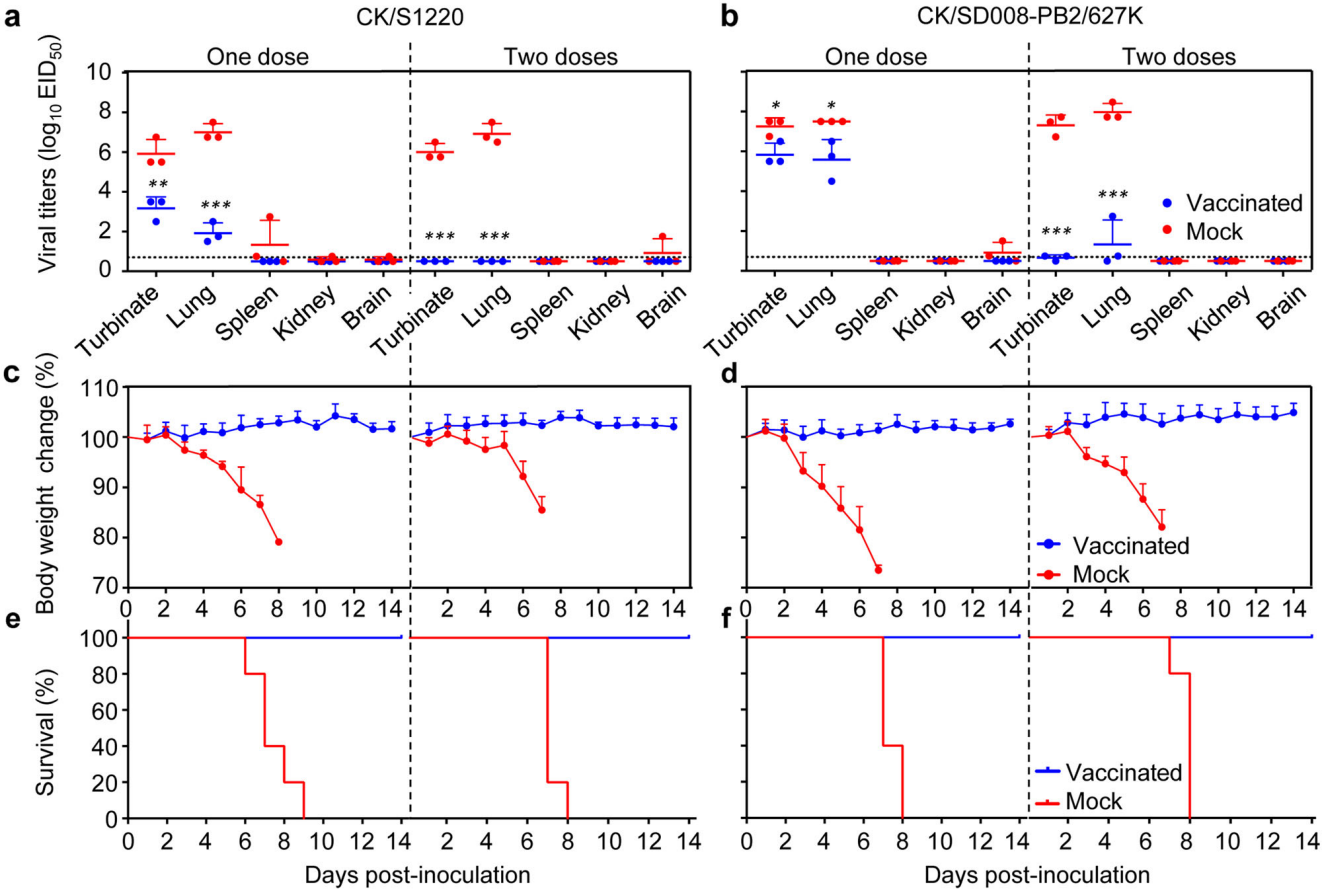
Protective efficacy of H7N9/AAca in mice against CK/S1220 and CK/SD008-PB2/627 K challenge. Groups of ten mice were vaccinated i.n. with 10^6^ EID_50_ of H7N9/AAca once or twice (three weeks apart); three weeks later, mice were challenged with the indicated viruses. Nasal turbinates, lungs, spleens, kidneys, and brains were collected from three mice in each group on day 3 post-challenge for virus titration (**a**, **b**). Body weight (**c**, **d**) and survival (**e**, **f**) of the remaining seven mice were monitored for 2 weeks. Data shown are the mean virus titers of three mice (**a**, **b**) or mean body weights of seven mice (**c**, **d**); the error bar shows the standard deviation. *P < 0.05, **P < 0.01, ***P < 0.001 compared with the corresponding value for PBS (mock)-vaccinated mice

In the single dose vaccinated groups, replication of the challenge viruses CK/S1220 and CK/SD008-PB2/627 K was detected in the nasal turbinates and lungs of mice, but the viral titers were significantly lower than those in the control groups (Fig. 2a, b). In the groups that received two doses of vaccine, replication of CK/S1220 was not detected in any of the organs tested, but CK/SD008-PB2/627 K was detected in the nasal turbinates and lungs of two out of three mice, and the viral titers were significantly lower than those in the control group (*p* < 0.001) (Fig. 2a, b). All of the mice in the vaccinated groups remained healthy and survived the CK/S1220 and CK/SD008-PB2/627 K challenges (Fig. 2c–f), whereas the mice in the PBS control groups experienced severe body weight loss and died within eight days of virus challenge (Fig. 2c–f). These results demonstrate that a single dose of the live attenuated H7N9/AAca vaccine protected mice from disease and death when challenged with different heterologous H7N9 highly pathogenic influenza viruses, but could not eliminate virus replication; however, after a second inoculation with the vaccine, replication of the challenge viruses was completely eliminated or reduced to a very low level.

### The CK/S1220 and CK/SD008-PB2/627 K viruses have different receptor-binding properties

**Fig. 3:**
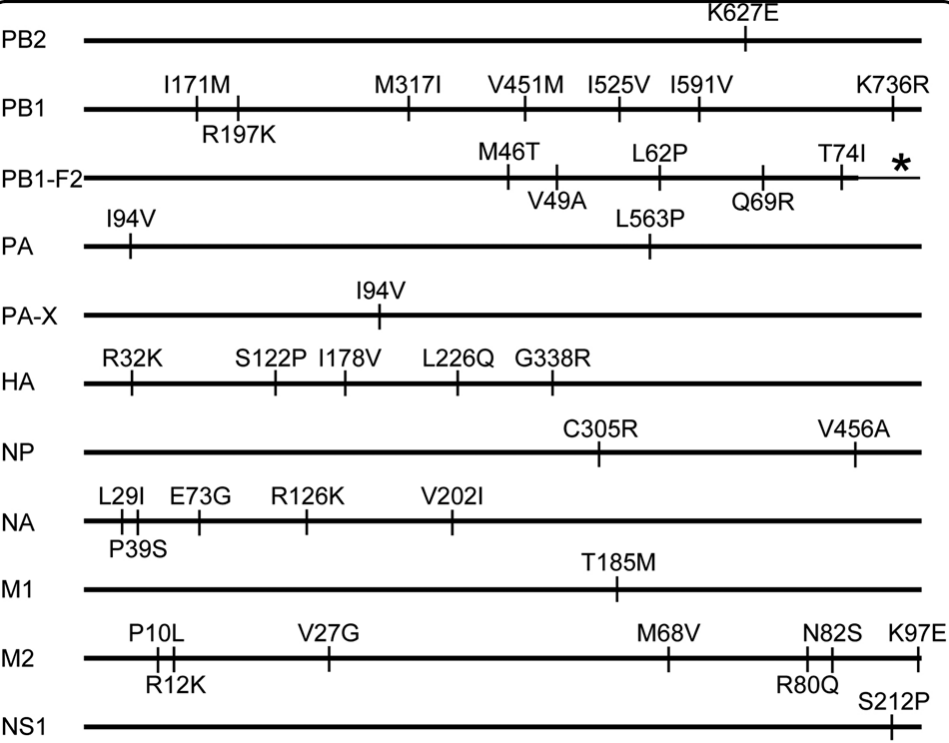
Amino acid differences between the CK/S1220 and CK/SD008-PB2/627 K viruses. The amino acid differences between the two viruses are shown as single letters at the indicated positions. Each amino acid of CK/SD008-PB2/627 K is shown before the number of the position, and each amino acid of CK/S1220 is shown after the number of the position. The amino acids at positions 32, 122, 178, and 226 of HA are in H3 numbering. *the PB1-F1 protein of CK/SD008-PB2/627 K has 14 more amino acids (-SKRWKLFSKQEWTN-) in its amino-terminal compared with that of the CK/S1220 virus

**Fig. 4:**
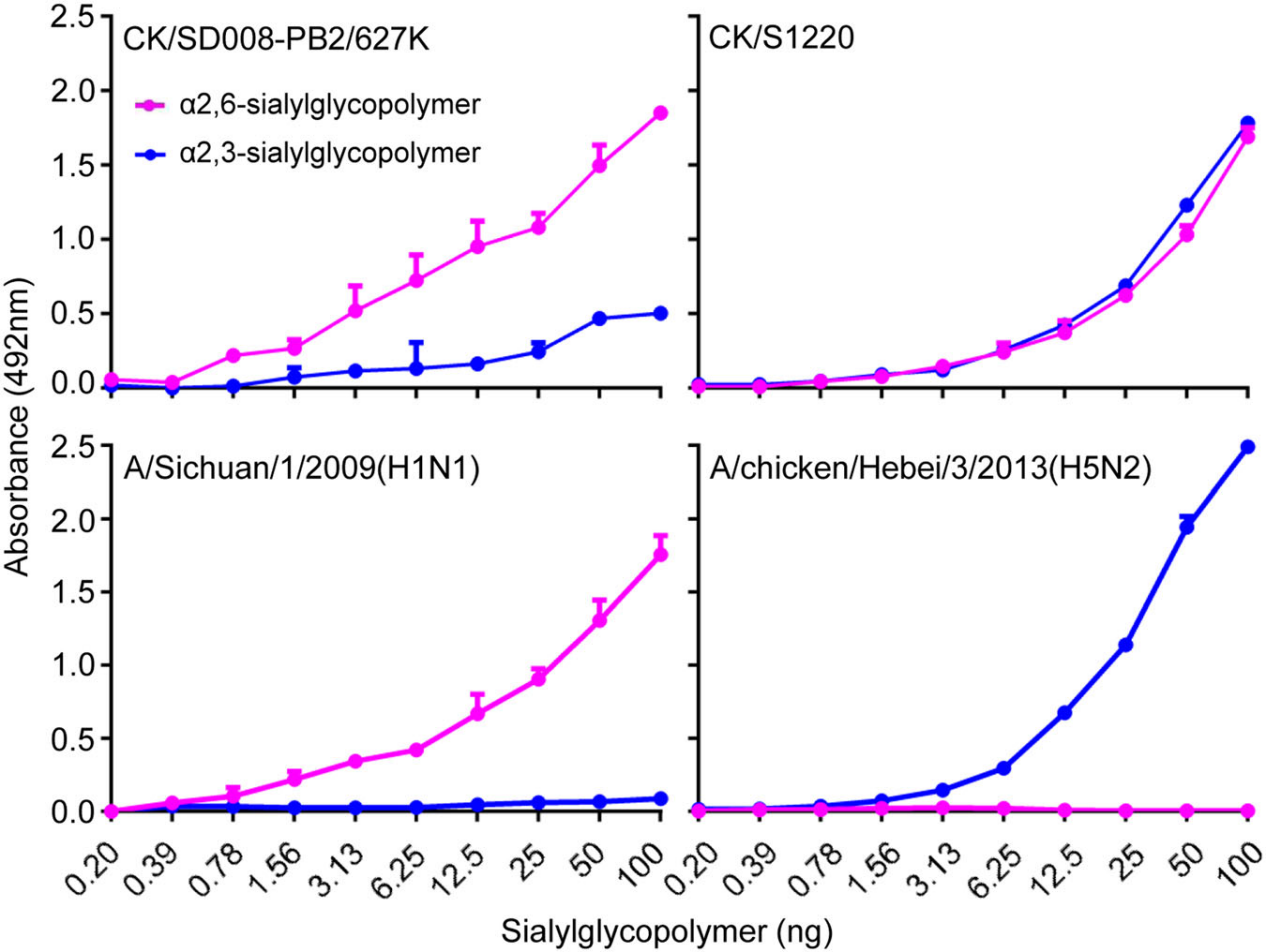
Receptor-binding properties of the CK/S1220 and CK/SD008-PB2/627 K viruses. Viruses were compared for their ability to bind to sialyglycopolymers containing either α2,6-siaylglycopolymer or α2,3-siaylglycopolymer. The A/Sichuan/1/2009(H1N1) and A/chicken/Hebei/3/2013(H5N2)viruses were used as controls

The CK/S1220 and CK/SD008-PB2/627 K viruses differ by 37 amino acids in their 11 proteins, and the PB1-F1 protein of CK/SD008-PB2/627 K has 14 more amino acids in its amino-terminal compared with that of the CK/S1220 virus (Fig. 3). The amino acids PB2 627 K and HA1226L (H3 numbering), which are reported to increase the replication and virulence of influenza virus in mammals and the affinity of H7N9 virus for human-type receptors, respectively^25,26^, are present in the CK/SD008-PB2/627 K virus, whereas the CK/S1220 virus carries PB2 627E and HA1 226Q. We tested the receptor-binding properties of CK/S1220 and CK/SD008-PB2/627 K as described previously^8,27,28^ and found that CK/SD008-PB2/627K bound to the α2, 6-siaylglycopolymer (human-type receptor) with higher affinity than that for the α2, 3-siaylglycopolymer (avian-type receptor), but CK/S1220 bound to theα−2, 3-siaylglycopolymer and α−2, 6-siaylglycopolymer with similar affinity (Fig. 4).

### The H7N9 highly pathogenic influenza virus CK/SD008-PB2/627 K transmits efficiently in guinea pigs via respiratory droplets

**Fig. 5:**
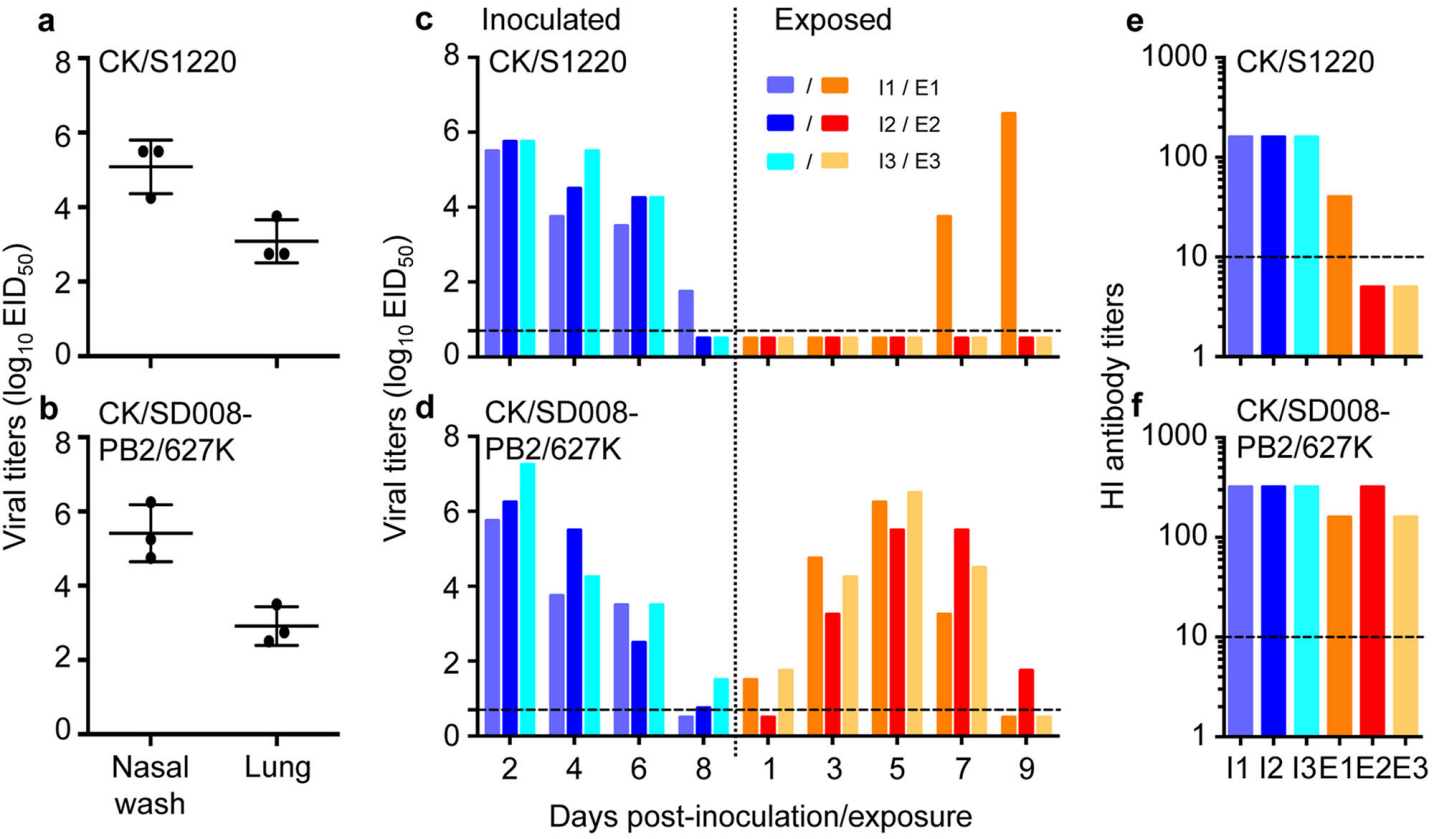
Replication and transmission of H7N9 highly pathogenic viruses in guinea pigs. To test for viral replication,groups of three guinea pigs were inoculated i.n. with 10^6^EID_50_ of the CK/S1220 (**a**) or CK/SD008-PB2/627 K (**b**) virus. The animals were killed on day 3 post-inoculation,and nasal washes and lungs were collected for virus titration. To test for transmission, groups of three guinea pigs were inoculated i.n. with 10^6^EID_50_ of the CK/S1220 (**c**) or CK/SD008-PB2/627K (**d**) virus, and each guinea pig was placed into a separate cage; 24 h later, three naive guinea pigs were placed in the neighboring cages. Nasal washes were collected every two days from all animals beginning 2 days p.i. to test for virus shedding. Seroconversion of the CK/S1220- (**e**) or CK/SD008-PB2/627K- (**f**) inoculated or exposed animals was tested for on day 21 post-inoculation or post-exposure. Each color bar represents the virus titer or antibody titer from an individual animal. The dashed lines in these panels indicate the lower limit of detection

Guinea pigs and ferrets are two commonly used animal models for evaluating influenza virus transmission^2,7,29–32^, but the transmission of certain influenza viruses differs between the two models^33^. We previously reported that CK/SD008-PB2/627 K was not only highly lethal in mice, but also efficiently transmitted in ferrets via respiratory droplets;^10^ however,whether H7N9 highly pathogenic influenza viruses transmit in guinea pigs is unknown. We therefore tested the replication and transmission of CK/S1220 and CK/SD008-PB2/627Kin guinea pigs. Groups of three guinea pigs were inoculated i.n. with 10^6^EID_50_ of virus, and the animals were killed on day 3 p.i. to collect their nasal washes and lungs for virus titration in eggs. As shown in Fig. 5, the two viruses replicated similarly: the mean viral titers of CK/S1220 in the nasal washes and lungs were 5.1log_10_EID_50_ and 3.1log_10_EID_50_, respectively (Fig. 5a), and those of CK/S1220 in the nasal washes and lungs were 5.4log_10_EID_50_ and 2.9log_10_EID_50_, respectively (Fig. 5b).

A transmission study in guinea pigs was performed by following a previously reported procedure^32^, which is briefly described in the Materials and Methods section. The CK/S1220 virus was detected in all of the three inoculated animals on days 2, 4, and 6 p.i., and in one animal on day 8 p.i. Virus was detected from one of the three exposed animals on days 7 and 9 post-exposure (p.e.) (Fig. 5c). All three inoculated animals and the one exposed animal that shed virus had seroconverted by day 21p.i. or p.e.(Fig. 5e). CK/SD008-PB2/627 K was detected from all three inoculated animals on days 2, 4, and 6 p.i., and in two animals on day 8 p.i. The virus was isolated from two of the three exposed animals on day 1 p.e. and from all three animals on days 3, 5, and 7 p.e. (Fig. 5d). All of the inoculated and exposed animals had seroconverted by day 21p.i. or p.e. (Fig. 5f). These results indicate that CK/S1220 and CK/SD008-PB2/627 K replicate similarly in guinea pigs, but differ in their transmissibility: CK/SD008-PB2/627 K was highly transmissible, whereas the transmission of CK/S1220 was inefficient. We therefore used CK/SD008-PB2/627 K as the test virus to evaluate the protective efficacy of the H7N9/AAca vaccine in preventing the transmission of H7N9 highly pathogenic virus.

### The H7N9/AAca vaccine prevents the transmission of CK/SD008-PB2/627 K in guinea pigs

Our above results indicated that even after two doses of the H7N9/AAca vaccine, low-level replication of the challenge virus CK/SD008-PB2/627 K was still detectable in the respiratory organs of mice (Fig. 2b). We then asked whether a single dose of the H7N9/AAca vaccine could prevent the transmission of the H7N9 highly pathogenic virus CK/SD008-PB2/627 K. We performed a transmission study to investigate (i) whether H7N9/AAca vaccination could prevent animals being infected upon expose to virus-inoculated animals, and (ii)whether H7N9/AAcavaccination could prevent infected animals from transmitting virus to naive exposed animals.

**Fig. 6:**
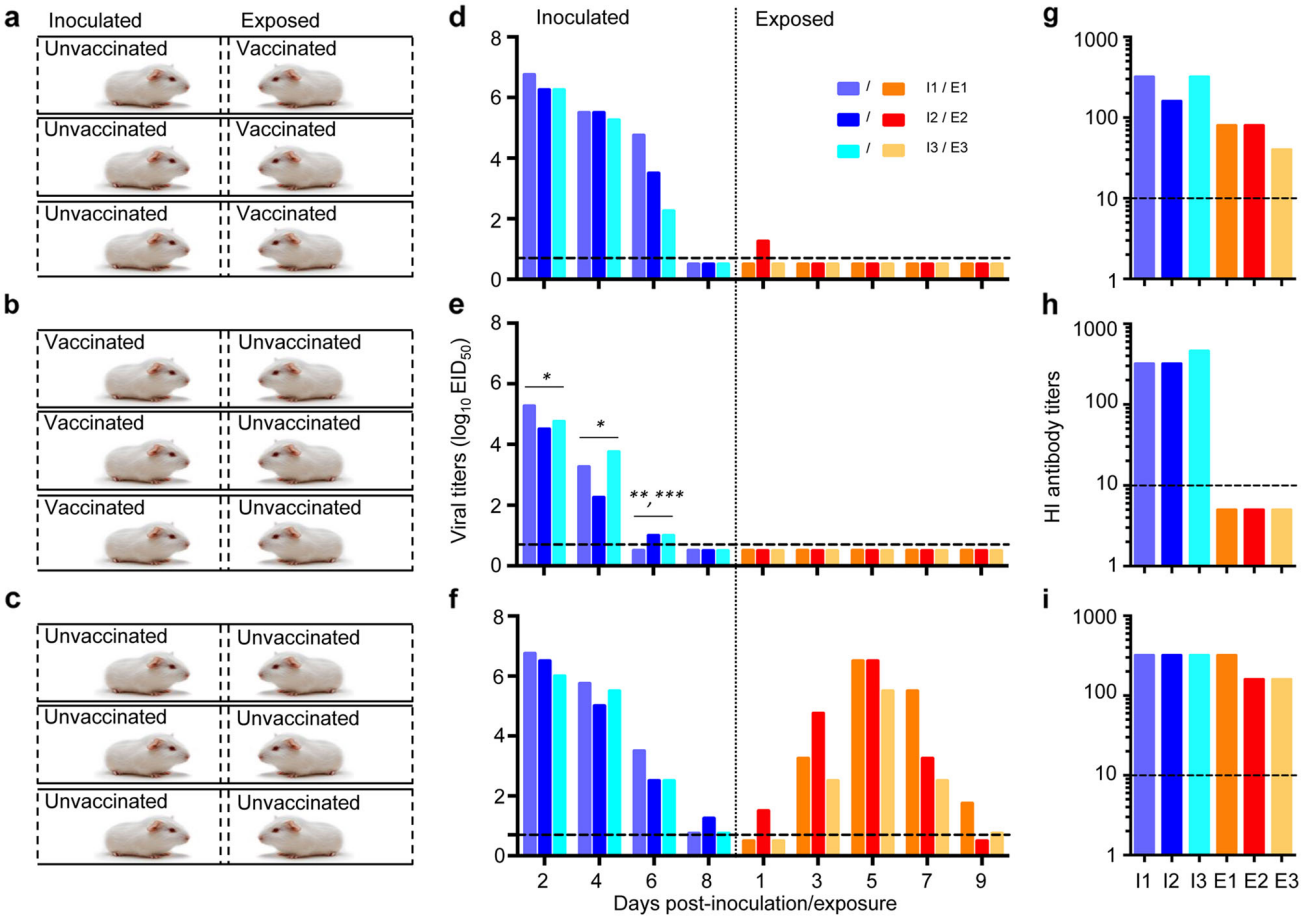
Effectiveness of the H7N9/AAca vaccine to prevent the transmission of H7N9 highly pathogenic virus in guinea pigs. Two groups of three guinea pigs were inoculated with 10^6^ EID_50_ of H7N9/AAca. Three weeks later, one group was put in the neighboring cages that hosted three guinea pigs that were inoculated with 10^6^.EID_50_ of CK/SD008-PB2/627 K virus (**a**); another group was inoculated with 10^6^ EID_50_ of CK/SD008-PB2/627 K virus; 24 h later, three naive guinea pigs were put in the neighboring cages (**b**). Transmission of the CK/SD008-PB2/627 K virus was also assessed in three pairs of unvaccinated animals as a control (**c**). The virus shedding titers of the animals in these three groups are shown in **d**, **e**, and **f**, respectively, and the HI antibody titers of the animals in these three groups are shown in **g**, **h**, and **i**, respectively. Each color bar represents a value from an individual animal. The dashed lines indicate the lower limit of detection.*P < 0.01 compared with the corresponding values in (**d**) and (**f**); **P < 0.05 compared with the corresponding values in (**d**), ***P < 0.01 compared with corresponding values in (**f**)

To address the first question, three guinea pigs were i.n. vaccinated with 10^6^EID_50_ of H7N9/AAca, and three weeks later, they were placed into three different cages next to animals that were inoculated 24 h before with 10^6^EID_50_ of the CK/SD008-PB2/627 K virus (Fig. 6a). To address the second question, three guinea pigs were i.n. vaccinated with 10^6^EID_50_ of H7N9/AAca, and three weeks later, these animals were inoculated with 10^6^EID_50_ of the CK/SD008-PB2/627 K virus and put into three separated cages; 24 h later, three naive guinea pigs were placed into neighboring cages (Fig. 6b). In addition, a transmission test of CK/SD008-PB2/627 K virus in 3 pairs of unvaccinated naïve animals was performed as a control (Fig. 6c). Nasal washes were collected from all of the animals every other day to check the virus infection.

In the CK/SD008-PB2/627 K virus directly inoculated groups, virus was detected in all of the animals (Fig. 6d–f), but the viral titers in the previously vaccinated animals were significantly lower than those in the unvaccinated animals (Fig. 6e). All of the CK/SD008-PB2/627 K virus directly inoculated animals seroconverted (Fig. 6g–i). In the exposed groups, low virus titers were detected on day 1 p.e. in one of the three exposed animals that were previously vaccinated with the H7N9/AAca virus (Fig. 6d), and all three of these animals seroconverted (Fig. 6g), which was induced by the AH/AAca inoculation rather than the CK/SD008-PB2/627 K exposure. Virus was not detected in any animals exposed to guinea pigs that were vaccinated with the H7N9/AAca virus and then inoculated with CK/SD008-PB2/627 K (Fig. 6e), and none of these animals seroconverted (Fig. 6h); virus was detected in all of the exposed animals in the control group (Fig. 6f), and all of these animals seroconverted (Fig. 6i). These results indicate that one dose of the H7N9/AAca virus vaccine could not prevent the replication of heterologous CK/SD008-PB2/627 K virus in guinea pigs but could efficiently prevent the transmission of the heterologous virus in guinea pigs.

## Discussion

Here, we evaluated the protective efficacy of the live attenuated H7N9/AAca vaccine against challenge with two different heterologous H7N9 highly pathogenic viruses (CK/SD1220 and CK/SD008-PB2/627 K) in mice. We found that one dose of H7N9/AAca protected mice from disease and death but did not proficiently prevent virus replication in the nasal turbinates and lungs of the mice. After two doses of the virus vaccine, the replication of CK/S1220 was completely eliminated, but a low titer of CK/SD008-PB2/627 K was still detectable in mice. These findings led us to test whether, and ultimately demonstrate that one dose of H7N9/AAca could prevent the transmission of CK/SD008-PB2/627 K in guinea pigs.

The replication of CK/S1220 in mice inoculated with two doses of H7N9/AAca was completely prevented, whereas that of CK/SD008-PB2/627 K in mice inoculated with two doses of H7N9/AAca was not completely eliminated, indicating that the H7N9/AAca vaccine provided better protection against CK/S1220 virus than against CK/SD008-PB2/627 K virus (Fig. 2).This difference in protection was likely not due to antigenic variation, because our serologic analysis showed that CK/SD008-PB2/627 K is antigenically closer to the vaccine strain than is CK/S1220. The difference may simply be the result of the CK/SD008-PB2/627 K virus replicating more rapidly than the CK/S1220 virus in mice.

Transmissibility of influenza virus is a polygenic trait. The CK/SD008-PB2/627 K virus transmits more efficiently than the CK/S1220 virus in guinea pigs via respiratory droplets.These two viruses differ by over 30 amino acids in multiple genes, and their PB1-F2 proteins differ in length. Although it is unknown how the genome difference affects the biologic properties of these two viruses, amino acid changes observed at two positions, namely PB2 E627K and HA1 Q226L, likely contributed to the difference in transmissibility of the two strains.

An important role of vaccination is to prevent virus transmission. We found that one dose of the H7N9/AAca vaccine did not prevent the replication of CK/SD008-PB2/627 K in guinea pigs, but significantly reduced the level of shedding of the challenge virus CK/SD008-PB2/627 K in guinea pigs and prevented its transmission to naive exposed guinea pigs. Our previous study showed that one dose of H7N9/AAca protected guinea pigs from infection when they were exposed to homologous AH/1 virus-inoculated animals^21^. In the present study, we found that when H7N9/AAca-vaccinated animals were exposed to CK/SD008-PB2/627K-infected animals, virus transmission occurred in one of the three pairs, but virus replication in the exposed animal was only detected on day 1p.e. and the virus was eliminated rapidly. It is highly unlikely that this low level of virus would have had the opportunity to transmit to other animals. These data therefore demonstrate that although one dose of H7N9/AAca vaccine could not completely prevent the replication of H7N9 highly pathogenic viruses, it may be able to prevent and eliminate virus transmission.

To control the H7N9 influenza viruses, the use of an H5/H7 bivalent inactivated vaccine in chickens was initiated in September 2017^34^. This vaccination campaign has played an important role in preventing H7N9 infections in poultry according to our surveillance data^24^. Moreover, the vaccination of poultry has essentially eliminated human infections with H7N9 virus in China: there were 738 human cases detected between 1 October 2016 and 10 June 2017, whereas only three H7N9 human cases have been detected since 1 October 2017. However, the H7N9 viruses have not been eradicated yet, and several outbreaks in chickens in northern China were reported in 2018;^35^ therefore, the risk of more human H7N9 infections and an influenza pandemic remains. The development and evaluation of effective human vaccines remains important for H7N9 influenza pandemic preparedness. Considering the advantages of live attenuated influenza vaccines compared with inactivated vaccines, as observed with other cold-adapted influenza live vaccines^17,21,36–40^, including easy production, easy application, broad protection against heterologous strains, and the protective efficacy in animals shown in our study, we suggest that H7N9/AAca has the potential to be an effective H7N9 vaccine and should be evaluated in humans. It is important to note that, to avoid the HA segment of the vaccine reassorting with human H1N1 or H3N2 viruses, which could lead to a pandemic, the live attenuated vaccine should only be used to contain an actual H7N9 pandemic, and not in pre-pandemic situations.

## Materials and methods

### Facility and ethics statements

All experiments with live H7N9 viruses were conducted within the enhanced animal biosafety level 3 (ABSL3 + ) facility in the Harbin Veterinary Research Institute (HVRI) of the Chinese Academy of Agricultural Sciences (CAAS) approved by the Ministry of Agriculture and Rural Affairs. This study was carried out in strict accordance with the recommendations in the Guide for the Care and Use of Laboratory Animals of the Ministry of Science and Technology of the People’s Republic of China.

### Vaccine and viruses

The live attenuated H7N9/AAca vaccine seed virus was generated by use of reverse genetics as reported previously^21^. The HA and NA genes were derived from the human isolate AH/1 and the six internal gene segments were derived from AAca. A/chicken/Hunan/S1220/2017 (CK/S1220) was an H7N9 highly pathogenic virus isolated from poultry in China (sequences were deposited in the GenBank with the accession numbers MH209304-209311)^24^. Our unpublished data showed that CK/S1120 is lethal for mice (MLD_50_ = 3.2log_10_EID_50_). CK/SD008-PB2/627K is a ferret-adapted virus derived from H7N9 highly pathogenic virus A/chicken/Guangdong/SD008/2017 (sequences were deposited in the GenBank with the accession numbers MF630034-630041), which was reported previously by Shi et al.^10^. Virus stocks were propagated in specific-pathogen-free (SPF) eggs and then kept at −70 °C before use in the animal studies.

### Mouse study

Groups of 10 six-week-old female BALB/c mice were anesthetized with CO_2_ and vaccinated intranasally (i.n.) with one or two doses (three weeks apart) of 10^6^ EID_50_ of H7N9/AAca in 50 µl or with 50 µl of PBS as a control. Sera were collected from 10 mice in each treatment group on day 21 post-vaccination (p.v.) for HI and NT antibody detection. The mice were then challenged i.n. with 100 MLD_50_ of the CK/S1220 (10^5.2^ EID_50_) or CK/SD008-PB2/627 K (10^3.8^ EID_50_) virus. Three mice in each group were killed on day 3 p.c. and their nasal turbinates, lungs, spleens, kidneys, and brains were collected for virus titration; the remaining seven mice in each group were observed daily for body weight change and death for two weeks.

### Receptor-binding analysis

Receptor specificity was analyzed by use of a solid-phase direct binding assay with two different glycopolymers:α2, 3-siaylglycopolymer[Neu5Acα2-3Gals1–4GlcNAcs1-pAP (para-aminophenyl)-alpha-polyglutamic acid (α-PGA)] (avian-type receptor)andα2, 6-sialylglycopolymer [Neu5Acα2–6Gals1–4GlcNAcs1-pAP (para-aminophenyl)-alpha-polyglutamic acid (a-PGA)] (human-type receptor) as described previously^27^. Chicken antisera against A/chicken/Shanghai/S1053/2013 (H7N9) virus, A/Sichuan/1/2009 (H1N1) virus, and A/chicken/Hebei/3/2013(H5N2) virus were generated in SPF chickens in our laboratory, and the horseradish peroxidase (HRP)-conjugated goat-anti-chicken antibody was purchased from Sigma-Aldrich(St. Louis, MO, USA).

### Guinea pig studies

Female guinea pigs (Vital River) weighing 250–280 g were used and were housed in cages placed inside isolators. To test the replication of CK/S1220 and CK/SD008-PB2/627 K in guinea pigs, groups of three guinea pigs were inoculated i.n. with 10^6^ EID_50_ of the test virus and killed on day 3 p.i.; their lungs and nasal washes were collected for virus titration in chicken eggs.

The transmission of CK/S1220 and CK/SD008-PB2/627 K in guinea pigs was studied by following the procedure that was previously reported by Zhang et al. and Wang et al.^28,32^. Briefly, groups of three animals were inoculated i.n. with 10^6^ EID_50_ of the test viruses in a 300-μl volume (150 μl per nostril). Twenty-four hours p.i., three naive animals were placed in adjacent cages as exposed groups; nasal washes were collected from exposed and inoculated animals at 2-day intervals for virus titration, beginning on day 2 p.i. (day 1 post-exposure (p.e.)); sera were collected on day 21p.i. or p.e. for HI antibody detection.

To evaluate the protective efficacy of the H7N9/AAca vaccine against the transmissibility of CK/SD008-PB2/627 K virus, we performed a transmission study as described above, but the inoculated or exposed animals were treated differently. Briefly, in the first group, the three exposed animals were vaccinated i.n. with 10^6^ EID_50_ of the H7N9/AAca vaccine in 300 μl three weeks before the day they were exposed to three guinea pigs that had been infected with 10^6^ EID_50_ of CK/SD008-PB2/627 K virus 24 h earlier (Fig. 6a). In the second group, the three guinea pigs in the inoculated group were vaccinated i.n. with 10^6^ EID_50_ of H7N9/AAca in 300 μl three weeks before they were infected with 10^6^ EID_50_ of CK/SD008-PB2/627 K virus (Fig. 6b). In the third group, neither the animals in the inoculated group or the exposed group were vaccinated (Fig. 6c). We collected nasal washes from all animals at 2-day intervals for virus titration in chicken eggs. All guinea pigs were killed on day 21 p.i. or p.e. for serum collection to test their HI antibody titers.

The ambient conditions were set at 20–22 °C and 30–40% relative humidity, and the airflow was horizontal with a speed of 0.1 m/s from inoculated animals to exposed animals.

### Antibody detection

In all antibody detection experiments, serum samples were treated with *vibrio cholera* receptor-destroying enzyme (DenkaSeiken) for 18 h at 37 °C, and heat-inactivated at 56 °C for 30 min. HI antibody titers were tested by using 0.5% (vol/vol) chicken erythrocytes. NT antibody titers were determined in chicken eggs. The cutoff value used for the HI and NT antibody assays was 10.

### Statistical analysis

Virus titers were compared by use of the Student’s *t*-test. Differences were considered significant when the *P* value was less than 0.05.

## References

[1] WHO. Influenza at the human-animal interface. http://www.who.int/influenza/human_animal_interface/h7n9/en/ (Accessed on 13 June 2018).

[2] Zhang Q (2013). H7N9 influenza viruses are transmissible in ferrets by respiratory droplet. Science.

[3] Belser JA (2013). Pathogenesis and transmission of avian influenza A (H7N9) virus in ferrets and mice. Nature.

[4] Richard M (2013). Limited airborne transmission of H7N9 influenza A virus between ferrets. Nature.

[5] Watanabe T (2013). Characterization of H7N9 influenza A viruses isolated from humans. Nature.

[6] Zhou J (2013). Biological features of novel avian influenza A (H7N9) virus. Nature.

[7] Gao Y (2009). Identification of amino acids in HA and PB2 critical for the transmission of H5N1 avian influenza viruses in a mammalian host. PLoS Pathog..

[8] Li X (2014). Genetics, receptor binding property, and transmissibility in mammals of naturally isolated H9N2 avian influenza viruses. PLoS Pathog..

[9] Qi, W. et al. Emergence and adaptation of a novel highly pathogenic H7N9 influenza virus in birds and humans from a 2013 human-infecting low-pathogenic ancestor. *J. Virol.* **92**, e00921–17 (2018). 10.1128/JVI.00921-17 PMC5752946 29070694

[10] Shi J (2017). H7N9 virulent mutants detected in chickens in China pose an increased threat to humans. Cell Res..

[11] Zhou L (2017). Preliminary epidemiology of human infections with highly pathogenic avian influenza A(H7N9) virus, China, 2017. Emerg. Infect. Dis..

[12] Yang, L. et al. genesis and spread of newly emerged highly pathogenic H7N9 avian viruses in mainland China. *J. Virol.* **91**, e01277–17 (2017). 10.1128/JVI.01277-17 PMC5686710 28956760

[13] Bart SA (2014). A cell culture-derived MF59-adjuvanted pandemic A/H7N9 vaccine is immunogenic in adults. Sci. Transl. Med.

[14] Chen Z (2014). Development of a high-yield live attenuated H7N9 influenza virus vaccine that provides protection against homologous and heterologous H7 wild-type viruses in ferrets. J. Virol..

[15] Bahl K (2017). Preclinical and clinical demonstration of immunogenicity by mRNA vaccines against H10N8 and H7N9 influenza viruses. Mol. Ther..

[16] Wong SS (2014). A single dose of whole inactivated H7N9 influenza vaccine confers protection from severe disease but not infection in ferrets. Vaccine.

[17] Chen H (2003). Generation and characterization of a cold-adapted influenza A H9N2 reassortant as a live pandemic influenza virus vaccine candidate. Vaccine.

[18] Smith GE (2013). Development of influenza H7N9 virus like particle (VLP) vaccine: homologous A/Anhui/1/2013 (H7N9) protection and heterologous A/chicken/Jalisco/CPA1/2012 (H7N3) cross-protection in vaccinated mice challenged with H7N9 virus. Vaccine.

[19] Joseph T (2008). A live attenuated cold-adapted influenza A H7N3 virus vaccine provides protection against homologous and heterologous H7 viruses in mice and ferrets. Virology.

[20] Leung HC (2015). An H5N1-based matrix protein 2 ectodomain tetrameric peptide vaccine provides cross-protection against lethal infection with H7N9 influenza virus. Emerg. Microbes Infect..

[21] Kong H (2015). A live attenuated vaccine prevents replication and transmission of H7N9 virus in mammals. Sci. Rep..

[22] Zhang F (2017). Human infections with recently-emerging highly pathogenic H7N9 avian influenza virus in China. J. Infect..

[23] Quan, C. et al. New threats from H7N9 influenza virus: spread and evolution of high- and low-pathogenicity variants with high genomic diversity in wave five. *J. Virol.* **92**, e00301–18 (2018). 10.1128/JVI.00301-18 PMC5952148 29563296

[24] Shi, J. et al. Rapid evolution of H7N9 highly pathogenic viruses that emerged in China in 2017. *Cell Host & Microbe ***24**, 1-11 (2018). 10.1016/j.chom.2018.08.006 PMC6310233 30269969

[25] Xiong X (2013). Receptor binding by an H7N9 influenza virus from humans. Nature.

[26] Zhang H (2014). The PB2 E627K mutation contributes to the high polymerase activity and enhanced replication of H7N9 influenza virus. J. General. Virol..

[27] Wang G (2014). H6 influenza viruses pose a potential threat to human health. J. Virol..

[28] Wang, Z. et al. A single-amino-acid substitution at position 225 in hemagglutinin alters the transmissibility of Eurasian avian-like H1N1 swine influenza virus in guinea pigs. *J. Virol.* **91**, e00800–17 (2017). 10.1128/JVI.00800-17 PMC5640871 28814518

[29] Herfst S (2012). Airborne transmission of influenza A/H5N1 virus between ferrets. Science.

[30] Lowen AC, Mubareka S, Tumpey TM, Garcia-Sastre A, Palese P (2006). The guinea pig as a transmission model for human influenza viruses. Proc. Natl Acad. Sci. USA.

[31] Yang H (2016). Prevalence, genetics, and transmissibility in ferrets of Eurasian avian-like H1N1 swine influenza viruses. Proc. Natl Acad. Sci. USA.

[32] Zhang Y (2013). H5N1 hybrid viruses bearing 2009/H1N1 virus genes transmit in guinea pigs by respiratory droplet. Science.

[33] Zhang Y (2012). Key molecular factors in hemagglutinin and PB2 contribute to efficient transmission of the 2009 H1N1 pandemic influenza virus. J. Virol..

[34] MoA. H7N9 recombinant vaccine will be used in chickens in China. http://www.moa.gov.cn/gk/yjgl_1/yjcl/ (Accessed on 19 June 2017).

[35] OIE. The H7N9 highly pathogenic viruses outbreak in Chickens in Shaanxi, Ningxia, Liaoning and Shanxi Province, China. http://www.oie.int/en/animal-health-in-the-world/update-on-avian-influenza/2018/ (Accessed on 13 June 2018).

[36] Maassab HF, Bryant ML (1999). The development of live attenuated cold-adapted influenza virus vaccine for humans. Rev. Med Virol..

[37] Cox RJ, Brokstad KA, Ogra P (2004). Influenza virus: immunity and vaccination strategies. Comparison of the immune response to inactivated and live, attenuated influenza vaccines.. Scand. J. Immunol..

[38] Fan S (2009). Immunogenicity and protective efficacy of a live attenuated H5N1 vaccine in nonhuman primates. PLoS Pathog..

[39] Shi J (2012). Protective efficacy of an H1N1 cold-adapted live vaccine against the 2009 pandemic H1N1, seasonal H1N1, and H5N1 influenza viruses in mice. Antivir. Res.

[40] Lee YJ (2016). Enhancement of the safety of live influenza vaccine by attenuating mutations from cold-adapted hemagglutinin. Virology.

